# Size Distribution Model and Development Characteristics of Corrosion Pits in Concrete under Two Curing Methods

**DOI:** 10.3390/ma12111846

**Published:** 2019-06-06

**Authors:** Qing Wu, Xuezhong Li, Jun Xu, Gang Wang, Wenhao Shi, Shilin Wang

**Affiliations:** 1 College of Civil Engineering and Architecture, Jiangsu University of Science and Technology, Zhenjiang 212000, China; wuqing@just.edu.cn (Q.W.); xujun@just.edu.cn (J.X.); wgstarsky@163.com (G.W.); 15751774639@163.com (W.S.); Shilin_Wang0602@126.com (S.W.); 2 School of Materials Science and Engineering, Southeast University, Nanjing 211189, China

**Keywords:** seawater sea-sand concrete, chloride ion, pitting, corrosion pitting distribution

## Abstract

In this paper, the effect of chloride ions on the development of corrosion pits in different reinforced concrete under different environmental conditions is studied. A fitting model for the size distribution of pits in seawater and sea-sand concrete (SSC) under different curing modes is established, and time-dependent fractal features are examined. Under wet/dry chloride cycles, the weight loss rate and corrosion rate of steel bars are higher, and the number of corrosion pits appears to increase on a small scale within 60-day. A majority of the corrosion is metastable pitting, and we propose a model to describe the size distribution of pitting in different periods. The good agreement between the proposed model and the available data illustrates that the proposed model is reliable and accurate. Macroscopic pitting occurs first in wet/dry chloride cycles. With the increase of concrete age, the size distribution of pits under wet/dry chloride cycles is uneven, and the pit sizes in submerged concrete tend to be equal.

## 1. Introduction

With the rapid development of China’s civil engineering industry, land and aggregate resources such as river sand are becoming increasingly scarce. Many coastal cities have begun to utilize sea sand as a construction material [1]. Chloride ions contained in marine aggregates pose a great threat to steel bars within concrete by destroying integrality of the steel bars’ passive film, resulting in corrosion of the steel bars and a significant reduction of service life of the structure [2,3,4,5,6].

Rusting of steel surfaces in reinforced concrete structures is mainly pit corrosion, and the development of corrosion pits lead to a loss of strength and ductility in steel bars [7,8,9,10,11]. At present, numerous studies on the development of corrosion pits related to chloride-induced corrosion are conducted. Darmawan et al. [12] established a time-varying model for the maximum pitting depth of steel bars in a chloride environment. For microscopic corrosion pits, domestic scholars draw on the corrosion theory of metal plates and metal pipes in the field of material science for analysis [13]. For analysis of pit size, some scholars use fractal theory to study the depth and diameter of corrosion pits in steel [14,15]. Stewart et al. [16,17] found that the maximum depth of corrosion pit on the surface of a steel bar is consistent with the Gumbel distribution. Pit shape on the steel strand is classified into pyramidal, ellipsoidal, and saddle-shaped. The three-dimensional sizes of the pits are analyzed using probability and fractal analysis. The two-dimensional model for cross-section of the largest corrosion pit is built, and the reduction of steel cross section is replaced by the pitting area [18,19]. This method can simplify the calculation process when the types of corrosion pits are relatively uniform.

Existing research on the corrosion of marine reinforcement concrete primarily focuses on the influence of pits on the mechanical properties of the steel [20,21,22,23]. In addition, common corrosion mechanism from chloride ions is not applicable here considering large amount of chloride already present on the surface of aggregates [24,25]. Chloride on the outside of aggregates is much different than chloride mixed into the aggregate in terms of combination, propagation mode, and efficiency [26]. Also, the influence of curing method on the microscopic distribution and development of corrosion pitting is rarely studied, so the specific development conditions of pitting on the surface of steel in concrete are still unknown. Therefore, it is necessary to build a time-dependent numerical model for the development of corrosion pits on the surface steel in seawater sea-sand concrete in different marine environments in order to reliably predict the durability of offshore reinforced concrete.

This paper explores the influence of curing method on the corrosion of steel in seawater and sea-sand concrete. The weight loss rate, corrosion rate, and number and size of surface corrosion pits in seawater sea-sand concrete under two curing modes are statistically analyzed. The relationship between rusting and microscopic corrosion pitting is examined and a time-dependent distribution function model for the size and quantity of pits under different curing methods is established using probability statistics. The development mechanism of pitting size in a chloride environment is clarified using fractal theory, and pit growth and evolution was analyzed.

## 2. Experimental Program

### 2.1. Preparation of Specimens

**Figure 1:**
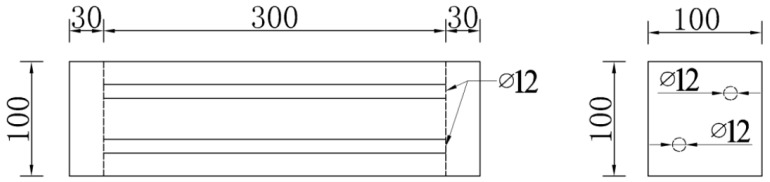
Sectional dimensions and reinforcement of specimens (Dimensions are in mm).

The strength grade of concrete used for specimens is designed as being able to bear 30 MPa, and the coarse aggregate is well-graded ordinary crushed stone and sea stone. The fine aggregate is natural medium sand and sea-sand, the fineness modulus $M_{x}$ is 2.8 and 2.65, and the chloride content of the sea-sand is 0.24%. The mix proportion is shown in Table 1. Ordinary concrete and sea-sand concrete samples have the following dimensions of 360 mm × 100 mm × 100 mm (Figure 1). Two 300 mm long and 12 mm diameter HPB300 steel bars are placed in a diagonal position. The concrete cover thickness is 20 mm.

### 2.2. Curing of Specimens

**Figure 2:**
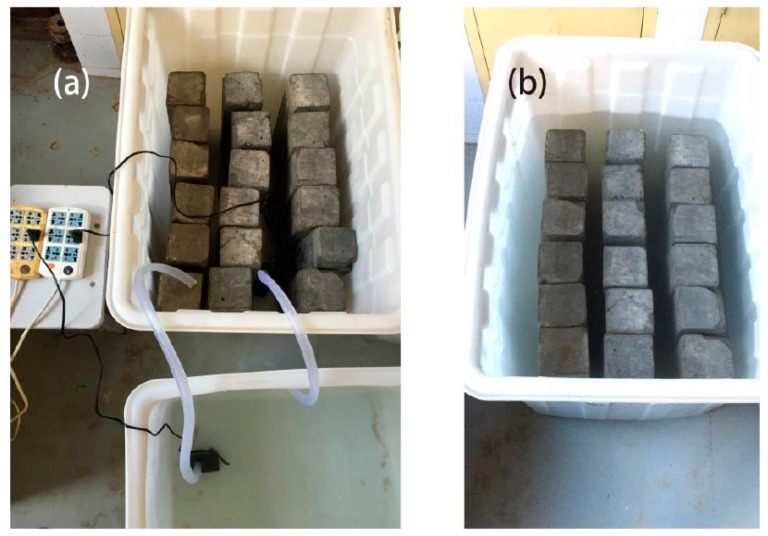
Curing methods of specimens; (**a**) schematic of the setup of the wet/dry cycles, (**b**) schematic of the setup of the immersion.

After the specimens are constructed, specimens are moved to the curing room and one group is continuously immersed in artificial seawater to simulate the marine environment. The other group was immersed in seawater in cycles to simulate the tidal environment of the ocean (Figure 2). Cycles included 2 days of submersion and 2 days of drying at room temperature (20 ± 5 °C) [27]. The artificial seawater’s preparation adopted the ratio in [28], and the water temperature is 20 ± 5 °C. Sample groups are shown in Table 2.

### 2.3. Measurement of Specimens

After the completion of chloride-induced corrosion tests in a simulated marine environment, samples were broken by mechanical press at 30-day, 60-day, and 90-day, and the embedded steel bars were removed. Samples are wrapped with glassine papers and rust spot contours are drawn. Sticking it and on the graph paper; the area of the rust can be counted out according to the statistical regulations. After pickling and rust removal, the steel bars were weighed to the nearest two decimal places. The metallographic microscope (ZEISS model, Oberkochen, Germany) was used to observe the surface of steel bars, and its image analysis system was used to carry out the size and quantity statistics of corrosion pits. The measurement includes three aspects: 1) rust spot area on the steel bars; 2) weight of the steel after pickling; 3) number and size of corrosion pits on the rebar surface, including the diameter (D) and area (S) of pits at different periods.

## 3. Results and Discussion

### 3.1. Steel Weight Loss Rate and Corrosion Rate

**Figure 3:**
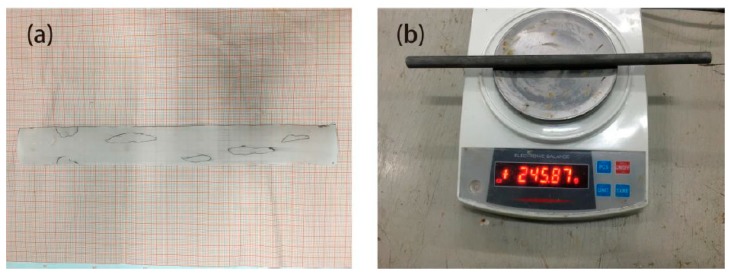
Statistics of corrosion rate and weight loss rate; (**a**) corrosion area statistics, (**b**) rebar quality statistics.

After completing the statistics, the percentage of corrosion area relative to the total surface area of the steel bar is calculated as the corrosion rate of the steel bars (Figure 3a). Results are shown in Table 3. The mass loss rate is computed to serve as a corrosion-degree indicator (Figure 3b). Results are shown in Table 4.

For seawater sea-sand concrete, steel bars began to corrode and produced large rust spots within 30-day, while in ordinary concrete rust spots are only present in the 90-day specimens, which is not enough to cause significant mass loss. The weight loss rate of steel bars in the wet/dry chloride cycle group is 33.33%, 44.89%, and 67.57% higher than that of the immersed samples separately. In the first 90 days of reinforced concrete maintenance, the average corrosion growth rate of steel bars under wet/dry chloride cycles is 0.096%/month, and the average rust growth rate of immersed steel bars is 0.061%/month.

The corrosion rate and the weight loss rate of samples are basically proportional. In seawater sea-sand concrete of the same age, the corrosion rate of steel bars in the wet/dry chloride cycle is 54.09%, 59.10%, and 45.55% higher than that of the immersed samples, confirming that corrosion is more serious when the oxygen is fully diffused.

### 3.2. Corrosion Pitting Size Statistics

The formation of etch pits generally goes through three stages: pitting nucleation, metastable micro-pits, and steady-state macroscopic pits [29]. The pitting nucleation stage is extremely unstable, and pit generation and decomposition on the metal surface are in dynamic equilibrium and are greatly affected by fluctuation of the potential difference in vicinity. In reinforced concrete structures, the concrete cover and strongly alkaline pore solution provide a stable environment for the generation and development of pitting nucleation. Pitting nucleuses are evenly distributed on the surface of the steel bar. Metastable micro-pit nucleation on the surface mainly depends on the geometry of the active point: the metastable micro-pits can be formed in the narrower, deeper active points, even at lower potential or in lower Cl^−^ concentrated solution. Shallower, more open active points must form metastable micro-pits at higher potential [30]. Pits produced in the metastable phase are also called microscopic pits. These pits are usually less than 0.2 mm in diameter and less than 0.15 mm in depth. Microscopic pits are mostly deep and narrow hemispherical pits, which are very small but visible to naked eye.

**Figure 4:**
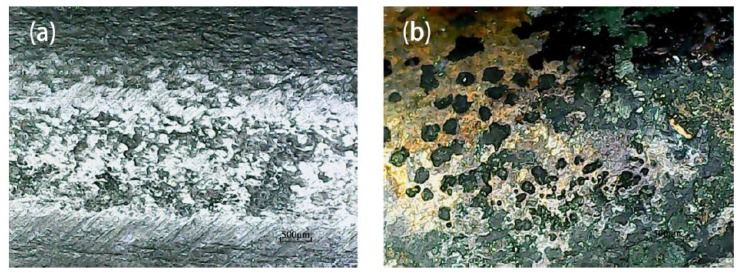
Contrast before and after corrosion; (**a**) steel surface before corrosion, (**b**) steel surface after corrosion.

Due to the production process of steel bars, the surface is not flat, and there are a large number of irregular pits. These irregular pits provide excellent environment for the generation of metastable micro-pits as well as growth and conversion to stable macroscopic pits (Figure 4a). After pickling, a large number of pits were densely and patchily distributed on the surface of the steel bar, and the distribution area was coincident with the position of the rust spot (Figure 4b). Corrosion pit diameter ranged from 0.02 mm to 0.17 mm, and the distribution was mostly within 0.1 mm to 0.15 mm. Statistics for pitting number and size distribution are shown in Table 5 and Table 6.

**Figure 5:**
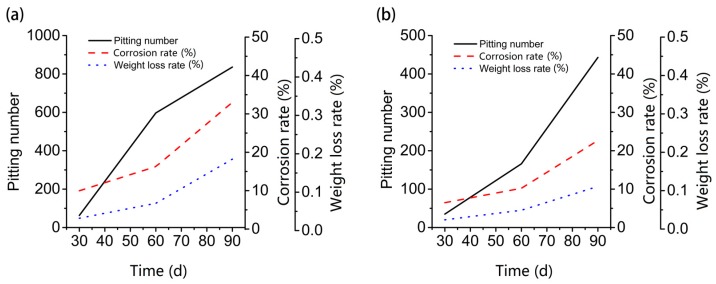
Comparison of the total number, corrosion rate, and weight loss rate; (**a**) contrast diagram of L1, (**b**) contrast diagram of L2.

**Figure 6:**
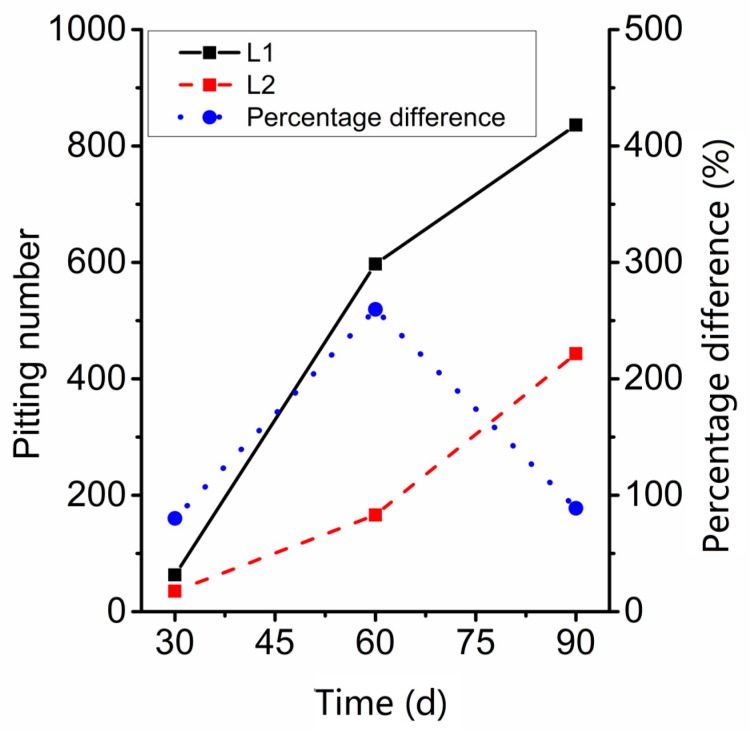
Contrast of the number of corrosion pits on L1 and L2.

For sample L2, the variation trend for total number of corrosion pits, corrosion rate, and weight loss rate are similar, and the growth rate increased steadily (Figure 5). At the same time, the growth rate of the corrosion rate and the weight loss rate for sample L1 increased gradually from 60-dat to 90-day, but the growth rate of the number of corrosion pits decreased at 90 days. When the corrosion rate and weight loss rate did not increase greatly, the total number of corrosion pits showed explosive growth from 30-day to 60-day. Considering the difference in the environment of the specimens, we speculate that with sufficient water and oxygen, corrosion pitting on the surface of steel will occur within30-day to 60-day, and then increase steadily. Similarly, from the trend of the number of pits in L1 and L2 (Figure 6), at 60-day, the percentage difference between L1 and L2 is the highest, which also proves that around 60-day, the corrosion pitting of steel in seawater sea-sand concrete under wet/dry chloride cycles reaches its higher rate.

### 3.3. Corrosion Pitting Size Distribution Model

**Figure 7:**
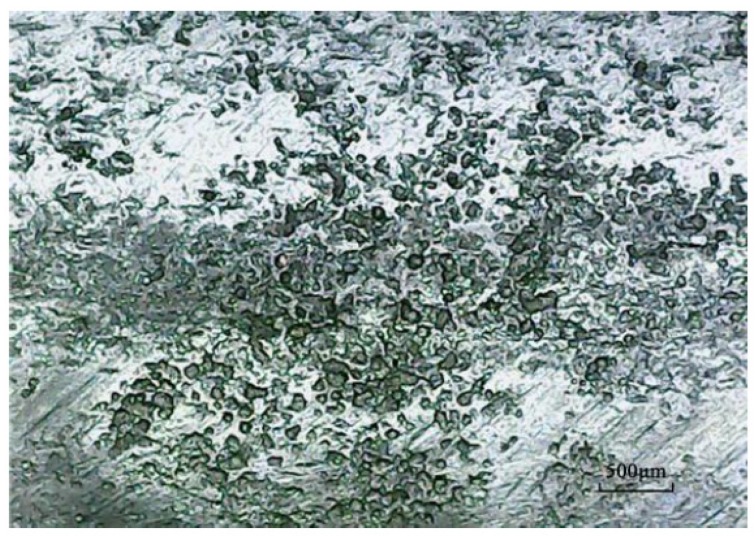
Typical corrosion pit on the surface of steel after pickling.

**Figure 8:**
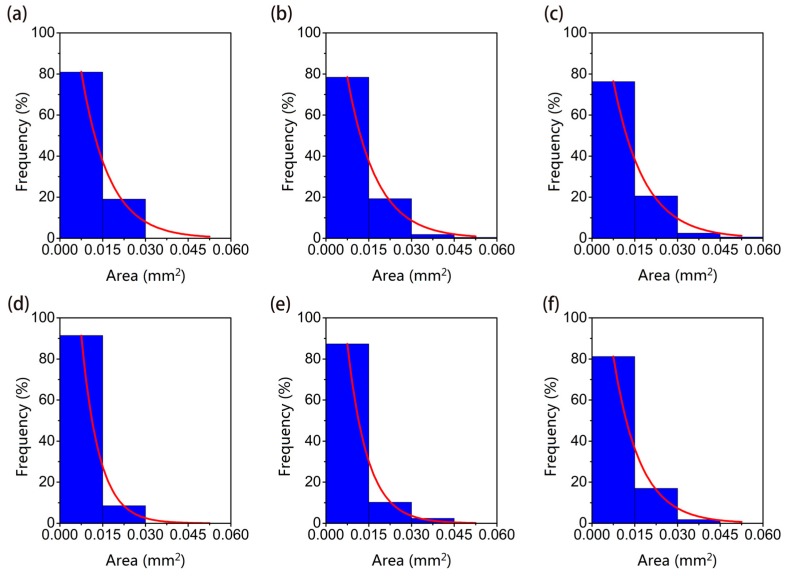
Probability distribution histogram of sizes of pits; (**a**) specimen L1, 30-day, (**b**) specimen L1, 60-day, (**c**) specimen L1, 90-day, (**d**) specimen L2, 30-day, (**e**) specimen L2, 60-day, (**f**) specimen L2, 90-day.

**Figure 9:**
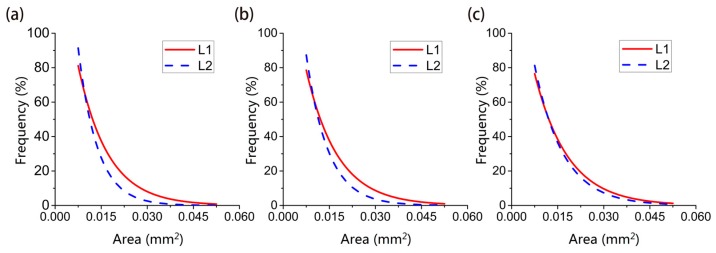
Comparison of fitting curve of probability distribution; (**a**) comparison of distribution curves 30-day, (**b**) comparison of distribution curves 60-day, (**c**) comparison of distribution curves of 90-day.

According to the classification in the specification, Corrosion pits are divided into seven types: narrow-deep, elliptical, wide-shallow, subcutaneous, undercut, horizontal, and vertical. Metastable micro-pits are mostly elliptical and wide-shallow pits obviously and can be regarded as having the same geometry (Figure 7). Statistically, pits from the two samples are grouped according to the following area intervals: (0, 0.015), (0.015, 0.03), (0.03, 0.045), (0.045, 0.06). The ratio of the number of pits to the total number in a given interval is taken as the appearance frequency f of the given interval. The appearance frequency f can be approximately equal to the appearance probability P. The frequency distribution of pitting size and the fitting curve of it related to the pitting size based on nonlinear least squares method in different periods is shown in Figure 8. The comparison of fitting curve between L1 and L2 in the same period is shown in Figure 9.

It can be seen from Figure 8 and Figure 9 that the time-dependent distribution of corrosion pits under the two curing conditions is as follows: Under the curing conditions of L1, although the total number of pits increased with time, the pits below 0.015 mm^2^ always accounted for approximately 80% of the total number of pits. The proportion of pits with an area between 0.015–0.03 mm^2^ was 20% and continued to grow. Metastable microscopic pits have been undergoing uninterrupted initiation and extinction due to fluctuations in corrosion currents. Although micro-pits with an area of more than 0.03 mm^2^ are less than 3% of the total number of pits, but these pits the key to the emergence of large-scale pits. Steel bar mechanical properties are determined by the development of these macroscopic pits.

Under the curing conditions of sample L2, the total number of pits and the number of pits in each interval are smaller than in sample L1, but the pitting distribution in each range is similar to that of L1. The distribution proportion of pits smaller than 0.015 mm^2^ reaches nearly 90%, and the pitting area of 0.015–0.03 mm^2^ are always kept at a level of ten percent approximately. It shows that for the same the surface chloride ion concentration and a lack of oxygen, corrosion pitting cannot fully develop.

We find that the probability distribution functions of pitting size in each period under both curing conditions are all exponential. Using the Levenberg-Marquardt algorithm, we propose a function model whose fitting function expression is:

(1) $$P=\alpha +\beta \times sin\left(\rho \times \pi \times t\times S\right)+\gamma \times exp\left(-{\left(\omega \times S\right)}^{2}\right)$$

where *α*, *β*, *ρ*, *γ* and *ω* are parameters, *P* is the probability of the pit distribution, *t* is the concrete age, and *S* is the area of the pitting.

According to the formula, we can calculate the pitting size distribution according to the maintenance method and maintenance time of the marine reinforced concrete. The specific corrosion condition of the steel bars in the concrete can be effectively deduced, and the durability of marine reinforced concrete can be predicted. The fitting results of the above functions and the goodness test are shown in Table 7.

### 3.4. Growth and Evolution of Corrosion Pits with Age for Two Curing Methods

Fractal is used to describe natural irregular behavior and complex physical phenomena [31]. The most basic feature of fractal theory is the quantitative description of the complexity and spatial filling ability of geometric shapes by means of fractal dimension perspectives and mathematical methods. In other words, the fractal and fractal dimension can be used to describe complex physical phenomena or dynamic processes. For samples with different corrosion times, the geometrical distribution characteristics of the pits can be compared by means of fractal theory.

A basic feature of the fractal system is that there is a negative power function relationship, that is, the dependent variable (corresponding the observation value) decreases rapidly with the increase of the independent variable (corresponding the measurement scale), and the two have a have a negative linear relationship in double logarithmic coordinates. The relationship is expressed as [32]:

(2) $$N\left(\varepsilon \right)\propto \varepsilon^{-D}$$

where ε is the dimension of the measurement unit; N(ε) is the number of elements measured at the scale of ε; D is the Hausdorff fractal dimension, which is a constant for a fractal system. Therefore, the definition of fractal dimension D is:

(3) $$D=\left[lnN\left(\varepsilon_{2}\right)-lnN\left(\varepsilon_{1}\right)\right]/-\left[ln\varepsilon_{2}-ln\varepsilon_{1}\right]$$

**Figure 10:**
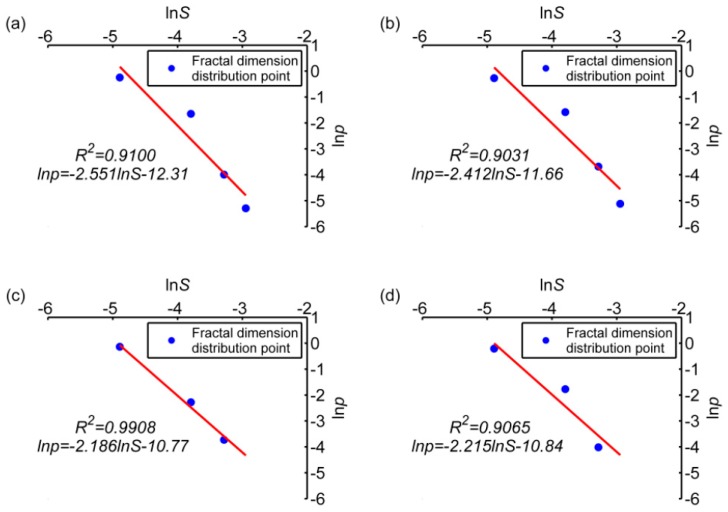
Fractal distribution characteristics of sizes of pits; (**a**) specimen L1, 60-day, (**b**) specimen L1, 90-day, (**c**) specimen L2, 60-day, (**d**) specimen L2, 90-day.

The pitting size distributions of samples L1 and L2 have obvious fractal features at 60-day and 90-day. At 30-day, due to the short corrosion time, the number of pits is small and not statistically significant. Figure 10 shows the scatter plot of area distribution; whose abscissa is the natural logarithm of the representative value (median value) of the corrosion pit area interval (area unit: mm^2^) and ordinate is the natural logarithm of the occurrence probability of the corrosion pits area interval in two periods. In this coordinate system, it is easy to see that most points have obvious linear features, and the results of linear fitting for these points are shown in Figure 10, too.

We can derive the fractal feature of these trend line distributions: The slope of the fitted polynomial graph is its fractal dimension. Under ideal uniform corrosion conditions, the size of the pits is also relatively uniform. The slope of the fitted graph should tend to be −∞, corresponding to D→∞. In the case of extreme uneven corrosion, the proportions of corrosion pits of different sizes is similar, and the slope of the fitted graph tends to be 0, corresponding to D→0.

The slope of the graph of the dry-wet cycle group is larger than that of the immersion group, indicating that the corrosion process of the dry-wet cycle group is more balanced than that of the immersion group (Figure 10). In other words, when macro-pits in L1 are somewhat developed, in sample L2 microscopic pits are just forming. In addition, with increasing time, the fractal dimension of L1 decreases slightly, indicating that the difference between pit sizes increases gradually, and the proportion of larger size pits increases gradually. Also, the fractal dimension of L2 increases slightly with time, indicating that pitting size is getting closer to the median value, and new corrosion pitting grows faster than old corrosion pitting.

In summary, from the beginning to 60-th day, the growth of old corrosion pits and the emergence of new corrosion pits are synchronized. From 60-day to 90-day, the corrosion protection effect on the pitted surface is weak, leading to well-developed old corrosion pitting. In addition, the emergence of new corrosion pits of L1 slows down. The difference between the numbers of corrosion pits of different sizes increases with time. By contrast, corrosion pitting development in sample L2 is very slow. In the early corrosion stage, new and old corrosion pits develop synchronously. The maximum pitting size in sample L2 increases, but the number of large pits is small, so the pitting size tends to the median overall. This relationship can also be analyzed from several conditions required for metal corrosion, first, oxygen more easily reaches the surface of the steel bars in the wet/dry cycle group through the capillary tubes in the concrete, allowing the metal at the pitting sites to be fully oxidized. Secondly, under dry-wet chloride cycles, the transport mode for chloride ions in concrete is mainly capillary absorption under a humidity gradient, which is more efficient than the diffusion under immersion conditions.

## 4. Conclusions

In order to search for the specific influence of chloride ions on steel in seawater sea-sand concrete in a marine environment, this paper examines the corrosion rate and weight loss rate of steel bars in concrete under two different curing methods and compares the total number of pits. The model of pit size distribution is established, and the growth and evolution mechanism of corrosion pitting is described according to the distribution of fractal features. The following conclusions are drawn:

(1) The steel in the ordinary C30 concrete will not corrode completely within three months under any environment, that is, chloride ions on the surface of the steel bar will not reach the critical corrosion concentration during this period. Chloride ions on the surface of the steel in seawater sea-sand concrete reached the critical corrosion concentration within 30 days, and the weight loss rate and corrosion rate developed steadily with time.

(2) Most corrosion pits on the steel surface in seawater sea-sand concrete are elliptical and have a wide-shallow shape. The number of pits increases in proportion to the weight loss rate and rust deposition rate growth. Furthermore, between 30-day and 60-day, the number of microscopic pits under wet/dry chloride cycles showed a small but rapid growth.

(3) The probability distribution model of pit size under two curing methods can be expressed as the function $P=\alpha +\beta \times sin\left(\rho \times \pi \times t\times S\right)+\gamma \times exp\left(-{\left(\omega \times S\right)}^{2}\right)$. About 90% of pits are metastable micro-pits in 90-day, and large-scale macroscopic pits first occur in the dry/wet chloride cycle group at 60-day, accounting for about 3%. 

(4) The size distribution of corrosion pits in seawater sea-sand concrete of different ages has obvious fractal features. In general, the development of pitting in the dry/wet cycle group is mainly based on the expansion of old corrosion pits, and the size difference gradually increases. Under the immersion condition, the sample is still in the stage of forming new pits, and the size is centralizing to the median. 

## Figures and Tables

**Table 1:** Concrete mix proportion (kg/m^3^).

Specimen Name	Types of Concrete	m-Cement	m-Water	m-Sand	m-Stone
L1	SSC	496.03	203.37	520.83	1279.76
L2	SSC	496.03	203.37	520.83	1279.76
L3	Ordinary concrete	496.03	203.37	520.83	1279.76
L4	Ordinary concrete	496.03	203.37	520.83	1279.76

**Table 2:** Grouping of reinforced concrete sample.

Specimen Name	Types of Concrete	Curing Methods	Exposure Period (d)		
L1	SSC	wet/dry cycles	30-day	60-day	90-day
L2	SSC	immersion	30-day	60-day	90-day
L3	Ordinary concrete	wet/dry cycles	30-day	60-day	90-day
L4	Ordinary concrete	immersion	30-day	60-day	90-day

SSC means seawater and sea-sand concrete.

**Table 3:** Corrosion rate of specimens.

Specimen Name	Types of Concrete	Curing Methods	Corrosion Rate (%)		
			30-day	60-day	90-day
L1	SSC	wet/dry cycles	9.97	16.26	33.01
L2	SSC	immersion	6.47	10.22	22.68
L3	Ordinary concrete	wet/dry cycles	0	0	3.22
L4	Ordinary concrete	immersion	0	0	0

**Table 4:** Steel corrosion weight loss rate of specimens.

Specimen Name	Types of Concrete	Curing Methods	Weight Loss Rate (%)		
			30-day	60-day	90-day
L1	SSC	wet/dry cycles	0.032	0.071	0.186
L2	SSC	immersion	0.024	0.049	0.111
L3	Ordinary concrete	wet/dry cycles	0	0	0
L4	Ordinary concrete	immersion	0	0	0

**Table 5:** Distribution statistics of pitting size of L1.

Specimen Name	Area Interval (/mm^2^)	Number of Pits		
		30-day	60-day	90-day
L1	0~0.015	51	468	638
	0.015~0.03	12	115	172
	0.03~0.045	0	11	21
	0.045~0.06	0	3	5
	Total	63	597	836

**Table 6:** Distribution statistics of pitting size of L2.

Specimen Name	Area Interval (/mm^2^)	Number of Pits		
		30-day	60-day	90-day
L2	0~0.015	32	145	315
	0.015~0.03	3	17	121
	0.03~0.045	0	4	7
	0.045~0.06	0	0	0
	Total	35	166	443

**Table 7:** Results and goodness of curve fitting.

Fitting Function	Function Type	*α*	*β*	*γ*	*ρ*	*ω*	SSE	R-Square
PL1	polynomial	0.00279	−0.01165	0.9359	0.4283	55.06	0.00106	0.9991
PL2	polynomial	0.00767	−0.03715	1.1350	0.6165	66.43	0.00522	0.9966

## References

[1] Zheng R., Yuan L., He Z. (2004). Sea Sand Corrosion to Reinforced Concrete and Countermeasure in Ningbo. Concrete.

[2] Ann K.Y., Ryou J.S. (2008). Variation in the Chloride Threshold Level for Steel Corrosion in Concrete Arising From Different Chloride Sources. Mag. Concr. Res..

[3] Xu J., Li F. (2017). A Meso-Scale Model for Analyzing the Chloride Diffusion of Concrete Subjected to External Stress. Constr. Build. Mater..

[4] Xu J., Li F. (2016). Analytical Model for Load Dependence of Chloride Penetration into Concrete. J. Mater. Civ. Eng..

[5] Jiang L., Liu R., Yang H., Mo L., Xu J. (2013). Influence of Chloride Salt Type On Critical Chloride Content of Reinforcement Corrosion in Concrete. Mag. Concr. Res..

[6] Han S., Joo H., Choi S., Heo I., Kim K.S., Seo S. (2019). Experimental Study On Shear Capacity of Reinforced Concrete Beams with Corroded Longitudinal Reinforcement. Materials.

[7] Cairns J., Plizzari G.A. (2005). Mechanical Properties of Corrosion-Damaged Reinforcement. ACI Mater. J..

[8] Stewart M.G., Suo Q. (2009). Extent of Spatially Variable Corrosion Damage as an Indicator of Strength and Time-Dependent Reliability of Rc Beams. Eng. Struct..

[9] Wang B. (2012). Failure Mechanism and Degradation Model of Rc Structure Corroded by Chloride Attack.

[10] Wang B., Yuan Y., Chen R. (2011). The Character and Evolution of Corrosion Pits On Steel Reinforcing Bar Surfaces Corroded by Chloride. J. China Univ. Min. Technol..

[11] Darmawan M.S. (2010). Pitting Corrosion Model for Reinforced Concrete Structures in a Chloride Environment. Mag. Concr. Res..

[12] Darmawan M.S., Stewart M.G. (2007). Spatial Time-Dependent Reliability Analysis of Corroding Pretensioned Prestressed Concrete Bridge Girders. Struct. Saf..

[13] Zhu L., Gu A., Liu H., Liu J., Ye X., Hu B. (2008). Study On Characters of Corrosion Advancing Edge of Typical High Strength Aluminum Alloys. J. Aeronaut. Mater..

[14] Bian L., Weng Y., Li X. (2005). Observation of Micro-Droplets On Metal Surface in Early Atmospheric Corrosion. Electrochem. Commun..

[15] Xu Y., Qian C., Bian L., Chen Y. (2012). Fractal Based Characterization of Nonuniform Corroded Surface Profile in Steel Bars. J. Basic Sci. Eng..

[16] Ma Y., Wang D. (2009). Research On Distribution Law of Maximum Corrosion Depth of Steel in the Chloride Environment. Sichuan Build. Sci..

[17] Stewart M.G., Al-Harthy A. (2008). Pitting Corrosion and Structural Reliability of Corroding Rc Structures: Experimental Data and Probabilistic Analysis. Reliab. Eng. Syst. Saf..

[18] Val D.V., Melchers R.E. (1997). Reliability of Deteriorating Rc Slab Bridges. J. Struct. Eng..

[19] Darmawan M.S., Stewart M.G. (2007). Effect of Pitting Corrosion On Capacity of Prestressing Wires. Mag. Concr. Res..

[20] Xu G., Zhang D., Liu D., Wei J. (2012). Research On Mechanical Properties of Corroded Steel Bar in Concrete Under Chloride Environment. J. Hydraul. Eng..

[21] Fan Y., Zhou J. (2003). Mechanical Property of Rusty Rebar Considering the Effects of Corrosion Pits. J. Build. Mater..

[22] Zhao G., He S., Ma Y., Cui J., Zhou F. (2016). Fragility Analysis of Offshore Isolated Bridge Based On Steel Pitting Corrosion Effect. China J. Highw. Transp..

[23] Wang B., Yuan Y., Li F., Du J. (2011). Deterioration Analysis of Yield Strength and its Probabilistic Model of Steel Bar Corroded by Chloride. J. Build. Mater..

[24] Xing F., Zhang X., Huo Y., Ding Z. (2008). Study On Corrosion Mechanism of Chloride Ion Permeating in Sands During Mixing to Mortar. J. Build. Mater..

[25] Xu J., Li F. (2018). Meso-Scale Analysis of Concrete Chloride Diffusion Considering Skins. ACI Mater. J..

[26] Ma H., Xing F., Dong B., Liu W., Huo Y. (2007). Study of Electrochemical Characteristics for Steel Corrosion in Sea Sand Concrete. Concrete.

[27] Xu X., Liu R., Yan Y., Li C. (2016). Effect of Wet and Dry Time Atioon Chloride Diffusion in Marine Concrete Structures. Bull. Chin. Ceram. Soc..

[28] Waters J.F. (2012). Measurement of Seawater Ph: A Theoretical and Analytical Investigation. Ph.D. Thesis.

[29] Szklarska-Smialowska Z. (1986). Pitting Corrosion of Metals.

[30] Burstein G.T., Liu C., Souto R.M., Vines S.P. (2004). Origins of Pitting Corrosion. Corros. Eng. Sci. Technol..

[31] Mandelbrot B.B. (1983). The Fractal Geometry of Nature.

[32] Zhang J. (1995). Fractal.

